# After 157 years, a second specimen and species of the phylogenetically enigmatic and previously monobasic genus *Nototylus* Gemminger & Harold, 1868 (Coleoptera, Carabidae, Nototylini)

**DOI:** 10.3897/zookeys.927.49584

**Published:** 2020-04-16

**Authors:** Terry L. Erwin, David H. Kavanaugh, David R. Maddison

**Affiliations:** 1 Hyper-diversity Group, Department of Entomology, MRC-187, National Museum of Natural History, Smithsonian Institution, Washington, P.O. Box 37012, DC 20013-7012, USA National Museum of Natural History, Smithsonian Institution Washington United States of America; 2 Curator Emeritus, Department of Entomology, California Academy of Sciences, 55 Music Concourse Drive, San Francisco, CA 94118, USA Californian Academy of Sciences San Francisco United States of America; 3 Department of Integrative Biology, 3029 Cordley Hall, Oregon State University, Corvallis, OR 97331, USA Oregon State University Corvallis United States of America

**Keywords:** Brazil, French Guiana, rainforest, antennal comb, Brasil, Guyana Francesa, bosque lluvioso, peine antenal, Brésil, Guyane, forêt tropicale, structure de toilette antennaire, Brasil, Guiana Francêsa, floresta tropical, pente antenal

## Abstract

The enigmatic beetle tribe Nototylini (Carabidae) is revised and a key to species is provided. Two species from South America are included in the genus. One species, *Nototylusfryi* (Schaum), is reviewed and a second, *Nototylusballi* Erwin & Kavanaugh, **sp. nov.**, is described as new. Each species is known from a single specimen, neither of which is in good condition. The possible function of what appears to be a unique antennal grooming structure on the front femur is discussed.

## Abstract (es)

La tribu enigmática de escarabajos Nototylini (Carabidae) se revisa y se provee una clave para las especies. Dos especies de América del Sur están incluidas bajo este género. Se revisa una especie, *Nototylusfryi* (Schaum), y una segunda, *Nototylusballi***sp. nov.**, se describe como nueva. Ambas especies se conocen de un solo espécimen, ninguno de los cuales está en buenas condiciones. Se discute la posible función de lo que aparece ser una estructura para acicalamiento antenal el fémur anterior.

## Abstract (fr)

Une révision taxonomique de la tribu énigmatique de coléoptères Nototylini (Carabidae) est présentée et une clé pour l’identification d’espèces est fournie. Deux espèces d’Amérique du Sud sont incluses dans le genre. Une espèce est examinée (*Nototylusfryi* (Schaum)) et, une deuxième est décrite comme nouvelle espèce (*Nototylusballi***sp. nov.**). Chaque espèce est connue à partir d’un seul spécimen dont aucun est en bon état. La possible fonction de ce qui semble être une structure de toilette antennaire sur le pro-fémur est discutée.

## Abstract (pt)

Uma enigmática tribo de besouros, Nototylini (Carabidae), é revisada, e uma chave para as espécies de *Nototylus* é fornecida. O gênero agora inclui duas espécies: *Nototylusfryi* (Schaum), aqui redescrita, e *Nototylusballi***sp. nov.**, descrita como nova. Ambas são conhecidas por somente um espécimen em mal estado de conservação. Discute-se a função de uma estrutura singular do femur anterior, possivelmente usada na escovagem da antena.

## Introduction

The affinities of the taxon *Nototylus* as described by Schaum in 1863 (under the preoccupied name *Tylonotus*) based on a single specimen have posed a conundrum for carabidologists since Schaum’s time. Complicating the interpretation of the form and structure of this unique specimen is the fact that its poor initial preservation has led to its almost complete disarticulation during subsequent studies (see Deuve 1994).

**Figures 1–8.**
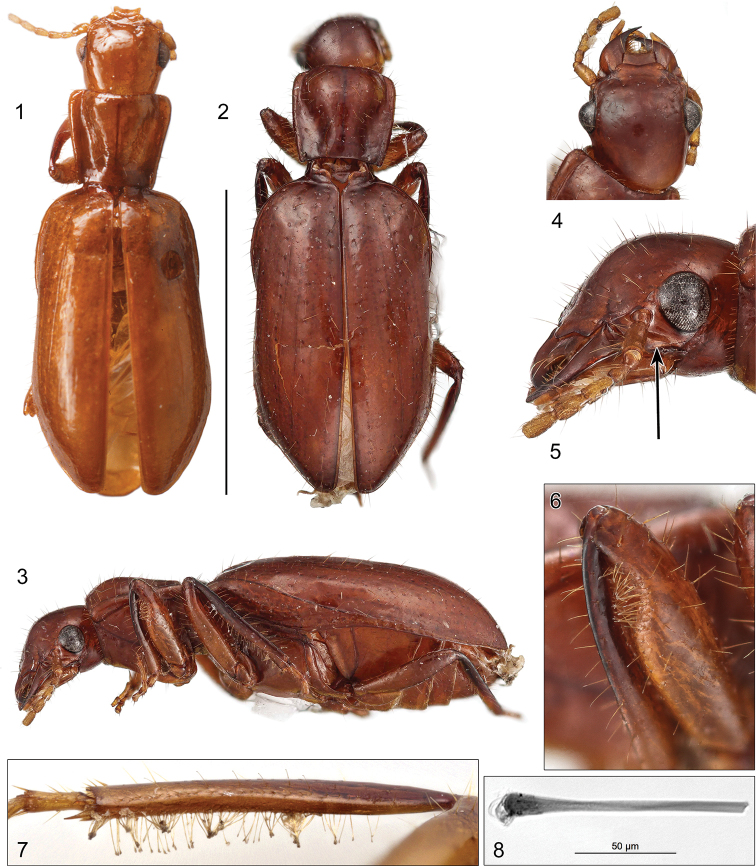
*Nototylus***1***Nototylusfryi* (Schaum), habitus, dorsal aspect, apparent body length (ABL) = 8.2 mm **2–6***Nototylusballi* sp. nov.: **2** habitus, dorsal aspect, ABL = 9.1 mm **3** habitus, left lateral aspect **4** head, dorsal aspect **5** head, left lateral aspect; arrow indicates location of sulcus beneath eye **6** left foreleg, lateral aspect; femur with antennal cleaner present subapicoventrally **7** left mesotibia, ventral aspect **8** closeup of a middle leg spatulate seta. Scale bars: 0.5 mm (**1–8**).

It has been reported that, unlike all other carabid beetles except highly evolved Paussini adults, adults of *Nototylusfryi* (Schaum), the type species of the genus, have no antennal cleaner on the anterior tibia, hence the origin of a long-standing debate about whether or not it belongs in the family Carabidae (Deuve 1994). Although Schaum’s original description made no mention of an antennal cleaner, nor was one shown in his illustrations, and Deuve (1994) made no mention of an antennal cleaner in his fine overall redescription, Erwin (1979, 2011) noted that the overall shape of the beetle is very ozaenine-like. Throughout their evolutionary history, carabid beetles have made sure that their antennae are kept clean, mainly through the development of combing structures (“antennal cleaners”) on the front legs, typically on the front tibiae. The selection pressure of ants on the Paussinae (including Ozaenini) and termites on taxa such as the Orthogonini has resulted in incredible transformations in carabid adult structures, so if *Nototylus* is another ant- or termite-associated group, then the “loss” of an antennal comb from the protibia would not be surprising (Fig. 1; Deuve 1994: fig. 11).

We here report on a second *Nototylus* specimen, one in somewhat better condition and representing a second species. This specimen, together with a re-examination of Schaum’s original specimen, permits us to report that there does indeed appear to be an antennal grooming structure present in *Nototylus* adults, but one in a different place and perhaps having a different function than is typical for a carabid. The purpose of this paper is to describe this new species and thereby confirm that the tribe Nototylini is still extant, at least in undecimated tropical forests in French Guiana. *Nototylusfryi* was described from the Brazilian State of Espíritu Santo, which is now mostly sugar cane fields, cacao plantations, and cattle ranches; and it has been considered that this species is likely now extinct (but see notes below).

## Materials and methods

This study is based on the examination of the only two *Nototylus* specimens known. Codens used in the text for institutions in which data or specimens are deposited (with names of curators in parentheses) are as follows:

**NHMUK**The Natural History Museum, London, United Kingdom (Beulah Garner);

**NMNH**National Museum of Natural History, Smithsonian Institution, Washington, DC, USA (Terry L. Erwin).

Methods and species concepts follow Erwin and Kavanaugh (1981) and Kavanaugh and Erwin (1991). The diagnosis and description format follow as closely as possible that suggested in Erwin and Johnson (2000). Measurements of length (ABL, SBL) and width (TW) follow those suggested by Ball (1972) and Kavanaugh (1979): ABL (apparent body length), measured from apex of labrum to apex of longer elytron; SBL (standardized body length), equals the sum of the lengths of the head (measured from apex of clypeus to a point on midline at level of the posterior edge of compound eyes), pronotum (measured from apical to basal margin along midline), and elytron length (measured from apex of scutellum to apex of the longer elytron); and TW, (total width), measured across both elytra at their widest point.

The images provided of the adult beetles described herein show most of the character states referred to in the description. The habitus images of the adult were made with a Visionary DigitalTM high resolution imaging system. Figures are all of the holotypes. The ADP number, which is a unique identification number for the specimen, links the specimen and associated illustrations and/or image to additional information in electronic databases at the National Museum of Natural History, Smithsonian Institution in Washington, DC (NMNH).

The photograph of a mesotibia and its setae were taken with a Leica Z6Apo lens and DMC4500 camera, and the close-up photograph of the setal apex with a Leica DM5500B compound microscope and DMC425C camera. Leica Application Suite v4.9 software was used to capture each image, and stacks of images from different focal positions were merged using the PMax procedure in Zerene Systems’ Zerene Stacker.

**Figure 9.**
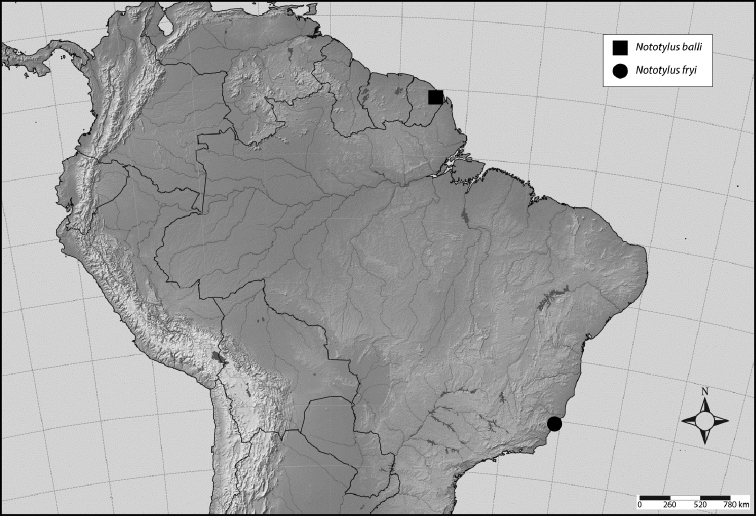
Map illustrating known distributions of species of *Nototylus*. Key: ■ = precise locality for *N.balli* sp. nov. (see text); ● = generalized locality for *N.fryi* (Schaum) in Brazil.

Geographical data of the new species were provided by the collector. A map (Fig. 9) indicates where the exact locale is in French Guiana. An English vernacular name also is proposed here because common names are becoming increasingly needed in conservation and/or agricultural and forestry applications.

## Taxonomic account

### Genus Nototylus

Taxon classificationAnimaliaColeopteraCarabidae

Gemminger & Harold, 1868

0F63823E-1274-5B8B-9133-FFCA420395CD

Tylonotus Schaum, 1863: 74 (preoccupied by Tylonotus Haldeman, 1847, a genus in the beetle family Cerambycidae, and Tylonotus Fieber, 1858 (Hemiptera).
Nototylus Gemminger & Harold, 1868: 161, new name.

#### Diagnostic combination.

Head domed, sub-hypognathus, with a partial sulcus (Fig. 5) under the anterior part of eye. Profemur with a subapicoventral concavity (Fig. 6) containing slender, elongate and apically ovospatulate setae (Fig. 8). This structure, unique within Carabidae, is presumed to be used for grooming the antenna (we will refer to it as a “grooming structure” below). Protibial antennal cleaner absent. Tibiae flattened as in carabids known to live with ants and lined with sparse apically spatulate setae. Deuve (1994) provided the following additional characteristics: procoxal cavities (*sensu*LeConte 1853) closed with pleural lobe fitted into the prosternal process; the harpalidian-type post abdomen (*sensu*Deuve 1988). Tergite IX differentiated as a thin transverse arch, laterotergites IX reduced and very lateral in location. The combination of character states in this enigmatic genus is unique within Carabidae.

#### Included species currently recognized.

*Nototylusfryi* (Schaum), 1863

*Nototylusballi* Erwin & Kavanaugh, new species

##### Key to adult females of *Nototylus* Gemminger & Harold, 1868

**Table:** 

1	Elytron (Fig. 1) only slightly swollen posterior to humerus; pronotum (Fig. 1) elongate, more strongly narrowed posteriorly, with lateral margins slightly sinuate, not rounded; brachypterous, hindwing without venation distal to stigma	***Nototylusfryi* (Schaum)**
–	Elytron (Fig. 2) more distinctly swollen posterior to humerus; pronotum (Fig. 2) subquadrate, with sides slightly rounded; macropterous	***Nototylusballi* Erwin & Kavanaugh, sp. nov.**


### Nototylus fryi

Taxon classificationAnimaliaColeopteraCarabidae

(Schaum), 1863

1DCA7A60-0409-50E7-BC61-B4FC693618AE

Fry’s strange-combed beetle Figures 1 , 9

Tylonotus fryi Schaum, 1863: 75.
Nototylus fryi (Schaum): Gemminger & Harold, 1868: 161.

#### Type material.

***Holotype*** female deposited in NHMUK. Detailed description and illustrations in Deuve (1994). See also Schaum (1863), Bänninger (1927), and Erwin (2011).

#### Geographical distribution

(Fig. 9). Known only from Brazil, Espíritu Santo, without precise locality.

#### Dispersal potential.

Brachypterous (wing truncated without distal venation), probably not capable of flight.

#### Way of life.

Unknown, except that they live in Southern Atlantic Forest (Mata Atlântica).

#### Note.

This taxon is known from a single disarticulated specimen in the NHMUK, which one of us (TLE) has re-examined twice. Its habitat, somewhere in the state of Espíritu Santo, Brazil, likely has suffered forest conversion to sugar cane, cacao plantations, or cattle ranches. The Bahia Coastal Forests ecoregion, which includes the state of Espíritu Santo, has less than 5% of the original forest vegetation remaining. See the web site http://www.worldwildlife.org/ecoregions/nt0103 (last accessed on 19 December 2019) for a very good description of what the area where this unique species lived was like previously and is like now (not good). Perhaps *N.fryi* is still extant in remaining protected areas such as Sooretama Biological Reserve and/or Linhares Forest Reserve. A major effort needs to be made to seek more specimens, particularly males, which remain unknown.

### Nototylus balli

Taxon classificationAnimaliaColeopteraCarabidae

Erwin & Kavanaugh sp. nov.

0448F28E-2165-5A75-AA0A-8186F0072E85

http://zoobank.org/2E9A04F6-AF58-43E2-8C79-7765690039A2

Ball’s strange-combed beetle Figures 2–8 , 9

#### Type material.

***Holotype***: A female, deposited in NMNH, labeled: French Guiana, Cayenne, track Bélizon, pk 4.5, 90 m (4.3704N, 52.3216W), July 2015 (JL Giuglaris) (NMNH: ADP143591, female).

#### Derivation of specific epithet.

The epithet, *balli*, is a Latinized eponym based on the family name of George E Ball, carabidologist and academic leader of a host of younger carabidologists, including all three coauthors, in celebration of his 90^th^ birthday, 25 September 2016. This species was introduced to George and many other carabidophiles at Athens, GA, during the Fourth International Symposium of Carabidologists in September 2016.

#### Diagnosis.

With the attributes of the genus as described above and slightly larger-sized than the *N.fryi* specimen. Adult with pale brown integument; only the mandibular apices and dorsal margins of tibiae infuscated. Head slightly broader and less narrowed posteriorly and with eyes more convex and hemispheric than in *N.fryi*. Frons and occiput moderately domed, aspect sub-hypognathus; smooth with fine, scattered setigerous punctures, perhaps with one slightly longer superorbital seta. Pronotum markedly domed, subquadrate, grossly explanate basolaterally, with lateral margins very slightly and evenly convex between front and hind angles (in *N.fryi*, lateral margins straighter and slightly sinuate anterior to hind angles); dorsum, margins and proepipleura sparsely setiferous. Elytron with humerus perfectly rounded, elytral silhouette more distinctly swollen posterior to humerus than in *N.fryi*, lateral margin markedly sinuate, disc markedly convex, apex at level of tucked post-femoral apex obliquely angulate, narrowly rounded apically to suture, not dentate, not plicate; lateral margin and epipleuron markedly setiferous. Interneurs with rounded or slightly elongate punctures, with uneven spacing between punctures. Hindwings macropterous.

#### Description.

(Fig. 2). ***Size***: ABL = 9.1 mm, SBL = 9.00 mm, TW = 4.6 mm. ***Color***: As described above. ***Luster***: Shiny. ***Head***: As described above. Antennae moderately short, filiform; antennal scape and flagellar antennomeres about twice as long as wide, length of pedicel slightly less than twice its width; all antennomeres with pubescence in addition to multiple fixed setae, with only sparse pubescence on scape and pedicel and denser pubescence on flagellar antennomeres. ***Prothorax***: Subquadrate, slightly constricted near base, hind angles produced posteriorly, anterior margin broader than neck; surface of disc as described above. ***Pterothorax***: Elytron markedly convex, slightly broader in anterior third with small epipleural flange, moderately flared from middle to apical third and rounded to hind angle; intervals flat, intervals 1, 3, and 5 with setigerous pores throughout length, interneurs striate. ***Legs***: Profemur with antennal grooming structure as described for genus (Fig. 6) and with protibia ventrally and mesotibia ventromedially (Fig. 7) with fringes of slender, elongate and apically ovospatulate setae (Fig. 8) like those in the profemoral grooming structure. ***Abdomen***: As described above. ***Male genitalia***: Unknown for this species. ***Female ovipositor***: see Deuve (1994) for *N.fryi*.

#### Note.

Based on unpublished scanning electron microscope images of the foreleg of the female holotype of *N.fryi* from George Ball and shared with us by Wendy Moore, we can now report that *N.fryi* also has the strange ovospatulate setae in the same locations as we have observed in the holotype of *N.balli*.

#### Geographic distribution.

(Fig. 9). This species is currently known only from the type locality in French Guiana.

#### Dispersal potential.

Macropterous and capable of flight. The holotype was collected with a glass pane flight intercept trap (FIT) (JL Giuglaris, pers. comm.).

#### Way of life.

Unknown, except that these beetles live in lowland rainforests and are extremely rarely collected. Only one specimen has been found so far despite the on-going beetle inventory by the Société entomologique Antilles-Guyane (SEAG) since 2014 in French Guiana (see Notes, below).

#### Notes.

The rainy season in French Guiana consists of heavy rainfall between December and July while the remaining months are comparatively dryer. Annual precipitation reaches 9.652 cm in and around Cayenne. Temperatures reach 25 to 27 °C as an average high at Cayenne. Thus, the specimen described herein was collected in the late warm rainy season a mere 57.4 km south of Cayenne.

From materials thus far collected by the SEAG inventory program, 19,272 carabid specimens have been sent to the first author, TLE. These specimens were collected from 30 different localities in French Guiana, mainly by FITs (flight intercept traps of both the glass pane and net types), but also at lights of various wave lengths (blue, pink), GEM lights, and SLAM traps (a small 4-sided malaise called the Sea, Land, and Air Malaise (SLAM) Trap that floats on water, stands on the ground, or hangs in the sky) (Erwin et al. 2012). None of those was a *Nototylus* specimen. An independent collector (not part of SEAG) came upon the single specimen described here, also collected in a glass pane FIT. It is unclear just why no adults of this species have been collected in any of the many SEAGFITs.

## Discussion

The setae in the profemoral grooming structure of the two specimens of *Nototylus* are unlike those seen in the protibial antennal cleaners of all other carabids. Here, they are long, slender, flexible, and apically ovospatulate (Fig. 8), whereas those of antennal cleaners of other carabids are stout, not or barely flexible, and sharply pointed apically. This suggests that the profemoral grooming structure seen here may have a somewhat different function than the protibial antennal cleaner of other carabids, the function of which is clearly seen when watching carabids groom themselves. The location of the profemoral structure in *Nototylus* is certainly one suitable for grooming the antenna; but the form and flexibility of these setae appear more suited to painting or coating the antenna than to scraping or cleaning it. Coupled with the occurrence of similar ovospatulate setae on the ventral surface of the protibia and the ventromedial surface of the mesotibia, it seems more likely that these setae function in applying or at least spreading some substance over the antennae and other parts of the body. The source of such a hypothetical substance is unknown, and we observed no structures, such as the variously located secretory trichomes seen in most if not all other carabids (and other beetles) that live with ants or termites.

These other colony “guests” use such substances to gain acceptance within the host colony. If our hypothesis that *Nototylus* live with ants or termites is correct, then the observed grooming structure and unusual setae may help to facilitate this symbiotic relationship. Clearly, we need to find and observe a living *Nototylus* adult to see how these structures are used.

## Conclusions

We had hoped, with this new specimen, to gather both molecular data and morphological data for nototyline male genitalia for the first time. Each of these data types could have led us to a better understanding of tribal relationships. Unfortunately, upon dissection the new specimen also turned out to be a female, as is the single known specimen of *N.fryi* (well-illustrated by Deuve 1994). Even more disappointing was the failure of our attempts to extract and sequence DNA from the specimen; these attempts will be reported in a separate publication. Consequently, we are no closer to understanding nototyline phylogenetic relationships than we have been for the last century and a half. At least we now know that this enigmatic group is still extant and that finding additional, fresh specimens, ideally even live specimens, is a real and most desirable possibility.

## Supplementary Material

XML Treatment for Nototylus

XML Treatment for Nototylus fryi

XML Treatment for Nototylus balli
